# Utility of low-profile visualized intraluminal support (LVIS™) stent for treatment of acutely ruptured bifurcation aneurysms: A single-center study

**DOI:** 10.3389/fneur.2023.1050369

**Published:** 2023-03-22

**Authors:** Changya Liu, Kaikai Guo, Xinxin Wu, Linguangjin Wu, Yike Cai, Xuebin Hu, BangJiang Fang

**Affiliations:** ^1^Department of Emergency, Longhua Hospital, Shanghai University of Traditional Chinese Medicine, Shanghai, China; ^2^Department of Neurosurgery, Union Hospital, Tongji Medical College, Huazhong University of Science and Technology, Wuhan, Hubei, China; ^3^Shanghai Skin Disease Hospital, Skin Disease Hospital of Tongji University, Shanghai, China; ^4^Emergency and Critical Care Institute of Shanghai University of Traditional Chinese Medicine, Shanghai, China

**Keywords:** stent-assisted coiling, ruptured aneurysms, bifurcation, low-profile visualized intraluminal support, LVIS

## Abstract

**Objective:**

Stent-assisted coiling has been increasingly used in the treatment of intracranial aneurysms. However, its application in ruptured bifurcation aneurysms remains controversial and challenging. This study aimed to present the safety and feasibility of low-profile visualized intraluminal support (LVIS™, LVIS, and LVIS Jr.) stent for acutely ruptured bifurcation aneurysms.

**Methods:**

A total of 41 patients with acutely ruptured intracranial aneurysms arising at the bifurcation were treated with LVIS™ stent-assisted coiling in our hospital between January 2017 and December 2021. The clinical data and angiographic results of the patients were analyzed.

**Results:**

Among these patients, all stents were successfully implanted. According to the immediate angiographic results, 29 aneurysms (70.7%) were completely occluded. Intraoperative thrombosis and hemorrhage occurred in two and one cases, respectively. No post-operative thrombosis or rebleeding events were observed. The clinical follow-up of all patients revealed that 38 (92.7%) cases had favorable outcomes (modified Rankin scale: 0–2). The angiographic results available for the 36 patients during the follow-up period revealed complete occlusion was achieved in 30 patients (83.3%) and residual neck in six patients.

**Conclusion:**

The LVIS™ stent-assistant coiling is a safe and feasible option for acutely ruptured bifurcation aneurysms. Further studies with a prospective design, a larger sample size, and long-term follow-up are needed to validate these findings.

## Introduction

The treatment goal for intracranial aneurysms is to reconstruct the morphological structure and restore the hemodynamics of the parent artery (1, 2). With the advances in minimally invasive techniques, endovascular treatment has emerged as a crucial treatment approach for managing intracranial aneurysms (3–5). The safety and effectiveness of stents have been assessed in cases with complex lesions and unruptured aneurysms (6, 7), such as those with wide necks, located distally, or of small size. However, the stent implementation for the management of acutely ruptured intracranial aneurysms situated at the bifurcation site remains debatable and poses a challenge, given the intricate anatomical structures comprising broad necks, the inclusion of vital branches, and diminutive vessels (8), such as anterior cerebral artery (ACA), anterior communicating artery (AcomA), middle cerebral artery (MCA), and basilar tip (9, 10). Furthermore, apprehensions have arisen regarding the potential for thromboembolic complications during stent deployment and the possibility of rebleeding in patients who have experienced subarachnoid hemorrhage and are undergoing dual-antiplatelet medication management (11).

The low-profile visualized intraluminal support (LVIS™, Microvention, Tustin, CA, USA) stent, which has two variations (LVIS and LVIS Jr.), is a recently developed self-expandable device that assists in the coiling process of intracranial aneurysms (12, 13). The braided structure provides high metal coverage and a smaller cell structure (14), which protects the aneurysm neck and important branch arteries. This structure affords stable support for density packing coils and good wall apposition to the parent artery, particularly in curved vessels (15). As a result, the utilization of this stent may augment the level of occlusion of the aneurysm and, in theory, lower the probability of aneurysm rebleeding (16–18). Recent scholars have documented some studies associated with the employment of LVIS stents for ruptured intracranial aneurysms and have considered LVIS stent-assisted coiling as an option for ruptured intracranial aneurysm endovascular management (19, 20). However, a limited amount of research is focused specifically on the implementation of LVIS for acutely ruptured aneurysms located at the bifurcation. Herein, we present a cohort of patients with acutely ruptured bifurcation aneurysms treated with LVIS stent-assisted coiling. We analyzed the clinical and angiographic data to determine the safety and feasibility of this therapeutic approach.

## Materials and methods

The institutional review board of our hospital approved this study (No. SOP-016-03-01), and informed consent has been obtained from all patients.

### Subjects

Surgery or endovascular procedures were chosen in interdisciplinary discussions for patients admitted to our institution with aneurysms. Endovascular treatment was the preferred procedure option, except for those who required open surgery or aneurysm clipping. For patients diagnosed with ruptured aneurysms, endovascular treatment was conducted promptly upon admission. Additionally, endovascular procedures in our present cohort were all performed within 3 days of disease onset. The therapy strategy was dependent on anatomical circumstances and the treating interventionalist.

**Figure 1:**
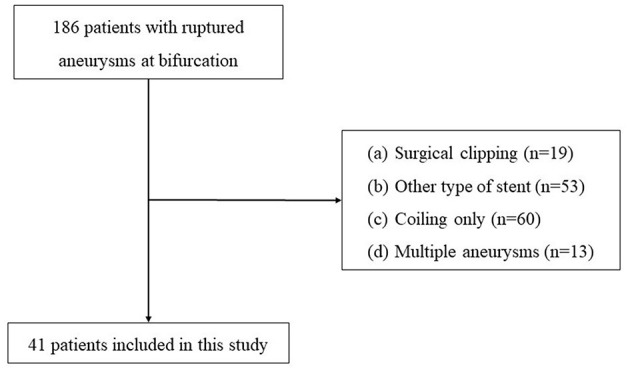
Flowchart for the recruitment process.

Between January 2017 and December 2021, 186 patients were admitted to our hospital with ruptured bifurcation aneurysms. We included patients based on the following exclusion criteria: patients (1) with blood blister-like aneurysms or multiple intracranial aneurysms; (2) who required double or multiple stents; (3) with a Hunt-Hess grade of IV–V before the procedure; (4) who received other embolization methods; and (5) who needed a craniectomy. Finally, a sample of 41 patients diagnosed with acutely ruptured aneurysms located at the bifurcation of the middle cerebral artery (MCA), anterior cerebral artery (ACA), anterior communicating artery (AcomA), or basilar tip and who underwent LVIS stent-assisted coiling was collected for the present study (Figure 1). In contrast, the other 145 patients, including 19 who received aneurysm clipping, 53 who received other types of stents, 60 who underwent coiling only, and 13 with multiple aneurysms who were also treated with other embolization methods, were excluded.

### Endovascular procedure

The endovascular treatment was performed for patients under general anesthesia. A bolus of 50 IU/kg of heparin was given and routinely administered during the procedure to achieve an activated clotting time of >250 s. The femoral artery was introduced with a 6 Fr short sheath (Terumo, Japan), and a 6 Fr guiding catheter (Envoy; Johnson & Johnson, USA) was advanced to the proximal arterial lesion to establish a pathway. The structure of the lesion was assessed by 3D digital subtraction angiography using a standard biplane machine (Artis Zee Biplane; Siemens, Germany). Then, a microcatheter (Headway; Microvention, CA, USA) was placed in the parent artery to deliver the LVIS stent. The decision between selecting the LVIS or LVIS Jr. is contingent upon a multifactorial assessment, taking into account the diameter of the parent artery as well as the clinical experience of the surgeon involved. The structural characteristics of these two variations are summarized in Table 1. Another microcatheter (Echelon-10; Medtronic, USA) was carefully introduced into the aneurysm sac. The semi-jailing technique (21) was applied to assist in the coiling packing density. The stent was fully deployed following the completion of the embolization procedure.

**Table 1:** Structural characteristics of the LVIS/LVIS Jr. stent.

	**Recommended vessel diameter (mm)**	**Microcatheter for delivery (inch)**	**Cell size (mm)**	**Radiopaque visualization structure**
LVIS	2.0 to 5.0	0.021	1.0	8 markers and 2 helical strands
LVIS Jr.	2.0 to 3.0	0.017	1.5	6 markers and 3 helical strands

### Antiplatelet therapy

Antiplatelet therapy was not prescribed before the operation. Furthermore, P2Y12 or other tests were not routinely used to measure individual responses to antiplatelet agents. When stent deployment was initiated, the patients were intravenously administered the glycoprotein IIb/IIIa inhibitor (Tirofiban, 100 ml/5 mg; Grand Pharma, Wuhan, China) at a dose of 0.10 μg/(kg/min) for 12 h. At the 9th h of infusion, a dosage of 75 mg clopidogrel and 100 mg aspirin was administered, either orally or *via* a nasogastric tube, daily for 3 months. Aspirin (100 mg/day) was maintained for at least 12 months. When post-procedural external ventricular drainage (EVD) was needed, surgical management was performed without discontinuing the antiplatelet medication.

For patients with intraoperative thrombus, as shown by stent thrombosis, slow blood flow in the parent artery during angiography, or the absence of distal arterial visualization, another microcatheter was employed, and intra-arterial tirofiban infusion was performed through the microcatheter, with the total dose of tirofiban not exceeding 1 mg.

### Evaluation of complications, angiographic results, and clinical outcomes

Incidents of perioperative hemorrhage and thromboembolic complications were documented. Intraoperative hemorrhage was defined as contrast extravasation from the aneurysm or parent artery during angiography. Post-operative rebleeding was defined as increased hemorrhage after the operation in computed tomography (CT). Intraoperative thromboembolism was determined by the manifestation of stent thrombosis, sluggish blood flow of the parent artery observed during angiography, or the absence of visualization of the distal arteries. Post-operative thromboembolism was delineated as novel symptoms or signs of thromboembolism that were corroborated by magnetic resonance or CT imaging.

The angiographic results were evaluated immediately after the operation and during the follow-up, using the Raymond-Roy scale: class I indicated complete occlusion, class II represented residual neck, and class III indicated dome filling. The clinical outcomes were assessed upon discharge and subsequently scheduled at 3, 6, and 12 months using the modified Rankin scale (mRS). Good clinical outcomes for the mRS scores were defined as scores ranging from 0 to 2, whereas poor clinical outcomes were categorized as scores ranging from 3 to 6. The clinical follow-up was evaluated by an outpatient interview. A 6-month angiographic follow-up was recommended, and each year after the operation, using digital subtraction angiography.

### Statistical analysis

The SPSS software version 22.0 (IBM SPSS Software, USA) was used for the statistical analysis. Continuous variables were presented as mean ± standard deviation, and categorical variables were presented in percentage. A *P*-value of < 0.05 was considered statistically significant.

## Results

Of the 41 patients, 24 (58.5%) were women, and the mean age of the entire cohort was 52.3 ± 8.9 years. Furthermore, 8 patients (19.5%) had combined hypertension, four (9.8%) patients were diagnosed with diabetes, and three patients had a history of smoking. Among these patients, 21 aneurysms were located at the MCA. Additionally, one aneurysm was found to be situated at the basilar tip and ACA, respectively. Furthermore, 18 aneurysms were observed to be located at the AcomA. The mean length diameter of these aneurysms was 5.4 ± 2.0 mm, and the mean neck width was 3.3 ± 0.6 mm. Prior to the operation, the Hunt-Hess scale scores indicated that 10 cases (24.4%) were classified as grade I, 17 cases (41.5%) were classified as grade II, and 14 cases (34.1%) were classified as grade III (Table 2).

**Table 2:** Baseline characteristics of the series of patients.

**Characteristics**	**Value**
**Gender**	
Male	17
Female	24
**Age**	52.3 ± 8.9
**Lesion location**	
Anterior communicating artery	18
Anterior cerebral artery	1
Middle cerebral artery	21
Basilar tip	1
**Aneurysm size (millimeter)**	
Length diameter	5.4 ± 2.0
Neck	3.3 ± 0.6
**Comorbid disease**	
Hypertension	8
Diabetes	4
**Smoking history**	3
**Clinical manifestation**	
Subarachnoid hemorrhage (SAH)	41
**Hunt-Hess grade**	
I	10
II	17
III	14

The data are presented in *n* (%) or mean ± standard deviation.

**Figure 2:**
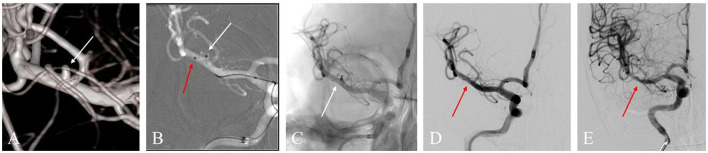
**(A)** A 53-year-old woman with a ruptured right middle cerebral artery (MCA) bifurcation aneurysm (white arrow) in the three-dimensional reconstruction image. **(B)** The roadmap image shows one sharp microcatheter catheterizing the aneurysm sac (white arrow) and another microcatheter placed in the parent artery (red arrow) for delivering astent device. **(C)** An LVIS Jr. stent (3.5 * 23 mm) was successfully delivered, and the aneurysm showed density packing from the coils (white arrow). **(D)** The final angiographic image manifested that the complete occlusion was achieved (red arrow). **(E)** DSA image follow-up in 12 months shows complete occlusion of the aneurysm (red arrow).

**Figure 3:**
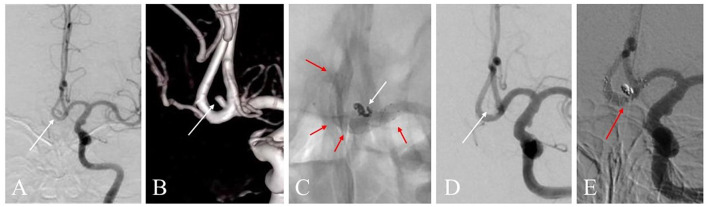
**(A)** A 59-year-old woman with a ruptured anterior communicating artery (AcomA) aneurysm treated with LVIS stent-assisted coiling strategy. Angiogram shows the AcomA aneurysm (white arrow). **(B)** Three-dimensional reconstruction imaging of the aneurysm (white arrow). **(C)** The LVIS stent (3.5 * 15 mm) was deployed during the procedure. The marks and the structure of the stent are presented (red arrow). The aneurysm showed density packing from the coils (white arrow). **(D)** Angiographic image shows the complete occlusion of the aneurysm (white arrow). **(E)** Digital subtraction angiography image of the aneurysm follow-up in 12 months shows the complete occlusion was achieved (red arrow).

The LVIS devices were successfully deployed in all 41 cases, including 33 LVIS stents and ten LVIS Jr. stents (illustrative cases are presented in Figures 2, 3). All procedures were completed. Intraoperative thromboembolism with in-stent thrombosis incidence was observed in two cases (4.9%), and this was successfully resolved by intra-arterial tirofiban infusion without the occurrence of associated neurological deficits after treatment. Intraoperative hemorrhage occurred in one case (2.4%), and this incidence was successfully managed by neutralizing heparin, rapidly packing small coils for dense embolization to achieve hemostasis, and finally deploying the stent successfully, without substantial neurological deterioration after treatment. Post-operative complications, such as thromboembolic or rebleeding events, were not observed. After the endovascular treatment, 18 patients received lumbar cisterna drainage, while 12 patients underwent lumbar puncture. None of the cases underwent an EVD procedure.

Following the immediate post-operative angiogram, 29 cases (70.7%) demonstrated complete occlusion (Raymond-Roy class I), 12 cases (29.3%) exhibited residual neck (Raymond-Roy class II), and no case was categorized as residual sac (Raymond-Roy class III). At discharge, 35 patients (85.4%) achieved mRS scores within the range of 0–2, four patients (9.8%) obtained a score of 3, and two patients (4.9%) scored 4.

Clinical follow-up data were obtained from all patients. Among these patients, 38 (92.7%) achieved good clinical outcomes with mRS scores of 0–2, while three (7.3%) had scores of 3. The angiographic follow-up data were available for 36 patients, with a mean follow-up time of 13.9 months. Among these patients, complete occlusion was achieved in 30 patients (83.3%), while an aneurysm neck remained in six patients. No recanalization was observed. In addition, no significant in-stent stenosis or parent artery occlusion was observed.

## Discussion

A total of 41 cases of acutely ruptured bifurcation aneurysms were treated with LVIS stent-assisted coiling in our cohort. All of the LVIS devices were completely deployed. Notably, 83.3% of the aneurysms were occluded completely, and 92.7% of cases had good clinical outcomes. These findings suggest that using the LVIS stent is safe and feasible for patients with acutely ruptured bifurcation aneurysms.

With the development of devices and techniques, such as remodeling balloons, laser-cut expandable stents, and multicatheter coiling techniques, endovascular procedures have been widely applied for managing intracranial aneurysms. However, considering the protection of branch arteries incorporated in the aneurysm base and sac, the rate of occlusion and recurrence, and the paradox of antiplatelet therapy with rebleeding risk remains a challenge when treating ruptured bifurcation aneurysms with stent-assisted coiling.

The augmentation of metallic coverage across the neck of an aneurysm through the use of a low-porosity structure potentially represents an effective approach to reducing blood flow within the aneurysm sac, promoting intra-aneurysmal thrombus formation, and facilitating vessel wall reconstruction. As such, the implementation of this approach may lead to better wall apposition, a heightened degree of immediate and subsequent aneurysm occlusion, and a decreased prevalence of both rebleeding and recurrence that might result in better outcomes (22). The LVIS stent has a relatively high surface metal coverage rate (23) when compared to laser-cut stents such as the Enterprise stent and Neuroform stent (24). In a study conducted by McEachern et al. (25), a total of 196 patients, including 21 ruptured aneurysms, received endovascular treatment with the LVIS Jr. stent, resulting in a long-term complete occlusion outcome for 85% of the cohort. Fiorella et al. (26) assessed the efficacy of the LVIS stent system in 153 non-acute onset patients and demonstrated a complete occlusion rate of 79.1% on angiographic outcomes at 12 months. In a retrospective analysis by Ge et al. (27), 190 patients with unruptured intracranial aneurysms who underwent stent implantation were assessed, wherein the LVIS stent group exhibited notably higher initial complete and near-complete obliteration rates in comparison to the Enterprise stent group (96.9%, 93/96 vs. 88.4%, 99/112; *P* = 0.034). Moreover, the angiographic follow-up revealed a lower recurrence rate in the LVIS stent cohort (2.8%, 1/36 vs. 10.7%, 6/56). Conversely, some studies have yielded disparate findings. Feng et al. (28) conducted a study with 142 patients, analyzing the occlusion status of aneurysms, and discovered no significant differences in angiographic outcomes between the LVIS stent group and the Enterprise stent group. However, logistic regression analysis indicated that the LVIS device may result in a lower rate of recanalization than the Enterprise stent. Zhang et al. (29) reviewed data from 56 studies published between 2015 and 2020 and reported comparable angiographic outcomes between the application of laser-cut and braided stents. However, they also found that the recurrence rate in the laser-cut stent cohort was higher than that of braided stents (6.87 vs. 5.52%). Recently, Mokin et al. (30) analyzed 659 patients, comparing the outcomes of endovascular management using Neuroform, Enterprise, and LVIS stents. The study presented significant differences in the complete occlusion rate on post-procedure imaging (LVIS 64.4%, 210/326; Neuroform 56.2%, 95/169; and Enterprise 47.6%, 68/143; *P* = 0.008) and follow-up imaging (LVIS 84%, 251/299; Neuroform 78%, 117/150; Enterprise 67%, 83/123; *P* = 0.004). In addition, their subgroup analysis for ruptured aneurysms revealed a higher complete occlusion outcome in the LVIS stent application group, including 76 aneurysms, compared to the laser-cut stent group at baseline (LVIS 80%, Neuroform 52%, and Enterprise 42%, *P* < 0.001) and follow-up (LVIS 86%, Neuroform 63%, and Enterprise 58%, *P* = 0.018). Unfortunately, their study did not distinguish the specific location of ruptured aneurysms. Consistent with previous research, our investigation of ruptured aneurysms located at bifurcations demonstrated a 70.7% complete occlusion rate in the immediate post-operative angiogram, with an 83.3% complete occlusion rate on angiographic follow-up. These findings indicate that the use of LVIS stent-assisted coiling is an effective approach for treating acutely onset bifurcation lesions.

The incidence of perioperative complications during the stent application for acutely ruptured aneurysms, specifically periprocedural thromboembolic complications and rebleeding while undergoing antiplatelet therapy, is a significant concern that hinders neurosurgeons from considering this treatment option. A previous study revealed that stent implanting in ruptured aneurysms arising at the location in small vessels beyond the circle of Willis may increase the rate of perioperative complications (31). Fan et al. (32) reported that the rate of perioperative bleeding and thrombus incidence was 9.5 and 15.9%, respectively, among 63 patients with ruptured aneurysms in the AcomA and treated with stent-assisted coiling. According to Zhou et al. (33), the procedure-related complication rate of stent implantation in the acute stage was 25.9%. These studies indicated the need for careful consideration of the benefits and drawbacks of stent implantation in managing acutely ruptured aneurysms. Furthermore, incomplete expansion of the stent in the lumen of the parent artery is a risk factor for periprocedural thromboembolic complications (17). Cho et al. (34) reported five of 27 (18.5%) patients with incomplete stent expansion during LVIS stent deployment. Poncyljusz et al. (35) reported the technical success rate of complete LVIS stent deployment as 91%.

The present study demonstrated 100% technical success with stent deployment, in which 4.9% (2/41) of cases had intraoperative thrombosis and 2.4% (1/41) of cases had an intraoperative hemorrhage. To the best of our knowledge, various factors may account for the low incidence of complications. Among these, the presence of a braided structure could offer some advantages for the management of complex aneurysms. The LVIS stents exhibit radiographic opacity and possess the capacity to be resheathed and repositioned, thus enabling convenient handling and accurate deployment. Second, the utilization of the “pull-push” technique during the stent deployment process, as well as the “bulging” technique, which entails the partial protrusion of the stent into the aneurysm's neck by pushing it across (36), provides good protection for both wide-necked aneurysms and their associated side-branches, facilitating improved attachment of the stent to the vessel wall. Third, the development of treatment materials such as the recently introduced more pliant coil materials may reduce the force applied to the aneurysm sac, potentially lowering the risk of rupture intraoperatively. Furthermore, the relatively low Hunt-Hess grade of our cohort and the use of intra-procedural cone-beam CT scans to monitor the stent expansion may contribute to a procedural facility in operation and improve clinical outcomes. In addition, the antiplatelet regimen and the usage of intravenous tirofiban in the present study, which were consistent with previous articles (37, 38), may be advantageous to the low rates of hemorrhage complications and thrombotic events.

In recent years, some novel devices have been developed for managing bifurcation aneurysms at the AcomA, MCA, and basilar tip, such as the Woven EndoBridge device (Sequent Medical, CA, USA), the PulseRider device (Pulsar Vascular, CA, USA), and the pCONus device (Phenox, Bochum, Germany) (39, 40). However, only the Woven EndoBridge device has been approved for clinical use in China but not for treating ruptured intracranial aneurysms. A prospective multicenter assessment of the Woven EndoBridge device in ruptured aneurysms conducted by Spelle et al. (41) presented a complete occlusion rate of 41.3% (19/46) at 1 year follow-up. Another study by Youssef et al. (42) revealed that 61.5% of cases achieved complete occlusion in follow-up. A systematic review conducted by Rooij et al. (43) revealed the rate of procedure-related complications in cases with ruptured intracranial aneurysms treated with the Woven EndoBridge device ranged between 1.8 and 27.3%, with the incidence rate of thromboembolic complications ranging between 1.8 and 21.0%. The follow-up occlusion rate ranged between 33.3 and 80.8%. Overall, the application of these new devices for ruptured intracranial aneurysms at the bifurcation warrants further evaluation.

The present study has some limitations. These included the retrospective and single-center design of the study and the relatively small sample size due to the highly selective cases treated with a relatively low Hunt-Hess grade, a specific stent, and an antiplatelet protocol. These may have introduced bias into the results.

## Conclusion

The present study reviewed 41 patients with acutely ruptured bifurcation aneurysms treated with LVIS stent-assistant coiling. The results revealed that the LVIS stent is a safe and feasible option for patients with ruptured bifurcation aneurysms, with a high complete occlusion rate and low complication incidence. Large-scale, multi-center investigations with longer follow-ups are needed to validate these present findings.

## Data availability statement

The original contributions presented in the study are included in the article/supplementary material, further inquiries can be directed to the corresponding authors.

## Ethics statement

The studies involving human participants were reviewed and approved by the Ethics Committee of Union Hospital, Tongji Medical College, Huazhong University of Science and Technology. The patients/participants provided their written informed consent to participate in this study.

## Author contributions

Conception and design and final approval of the version to be published: BF, XH, and CL. Analysis and interpretation of the data and review of the submitted version of the manuscript: CL and XW. Drafted the article: CL and KG. Critically revised the article: BF and XH. Statistical analysis: LW and XW. Administrative, technical, and material support: LW, YC, and KG. All authors contributed to the article and approved the submitted version.
